# Trends in statin utilization and ischemic heart disease mortality in Lithuania and Sweden, 2000–2020

**DOI:** 10.48101/ujms.v129.10412

**Published:** 2024-05-15

**Authors:** Indre Treciokiene, Kamile Daukintyte, Paul Hjemdahl, Björn Wettermark

**Affiliations:** a Pharmacy and Pharmacology Centre, Faculty of Medicine, Vilnius University, Vilnius, Lithuania; b Department of Medicine Solna, Clinical Epidemiology Unit, Karolinska Institute and Clinical Pharmacology, Karolinska University Hospital, Stockholm, Sweden; c Department of Pharmacy, Faculty of Pharmacy, Uppsala University, Uppsala, Sweden

**Keywords:** drug utilization, lipid-lowering drugs, ischemic heart disease, Lithuania, Sweden

## Abstract

**Aims:**

To compare statin utilization and ischemic heart disease (IHD) mortality trends in Lithuania and Sweden and to assess correlations between the total utilization of statins and IHD mortality.

**Methods:**

An ecological study assessing time trends in statin utilization (DDDs per 1000 inhabitants per day; DDD/TID) and IHD mortality in Lithuania and Sweden between 2000 and 2020. Statin utilization data in Lithuania were wholesale trade data, and Swedish data were drugs dispensed at pharmacies. IHD mortality data were extracted from national databases as rates per 100 000 inhabitants. Associations between statin utilization and IHD mortality in Lithuania and Sweden were examined using Spearman’s rank and Pearson’s correlation coefficients, respectively.

**Results:**

Statin utilization increased from 16.8 to 135.8 DDD/TID in Sweden and from 0.2 to 61.8 DDD/TID in Lithuania between 2000 and 2020. Medium intensity was the most common statin dosage in Lithuania, while Sweden used more high intensity than moderate-intensity statins from 2017. IHD mortality in Lithuania remained high between 2000 and 2020 (from 359.1 to 508.8 deaths per 100 000 population), while it decreased markedly in Sweden (from 226.87 to 88.7 deaths per 100 000 population). IHD mortality and statin utilization were inversely correlated in Sweden (r = -0.993, *P* < 0.001), while a positive correlation was found in Lithuania (rs = 0.871, *P* < 0.001).

**Conclusion:**

Despite the growing statin utilization in both countries, Lithuania recorded a slight increase in IHD mortality rates unlike the situation in Sweden. This indicates room for improvement in the management of modifiable cardiovascular risk factors in Lithuania including how statins are prescribed and used in clinical practice.

## Introduction

Cardiovascular diseases (CVDs) are the leading cause of death globally, attributable to more than 4 million deaths annually in Europe (1, 2). Ischemic heart disease (IHD) is the most common single cause of death, resulting in 19% of deaths in men and 20% of deaths in women. However, there have been substantial decreases in CVD and IHD mortality over the last decade – although to varying degrees in different countries (1, 2). In 2017, Lithuania still had substantially higher age-standardized IHD death rates in men and women compared to Sweden (3).

Behavioral risk factors such as unhealthy diet, lack of physical activity, tobacco and alcohol use, high blood pressure, elevated blood glucose and lipids, and overweight and obesity constitute the most important risk factors behind the development of IHD and stroke (1). Clinical and genetic evidence has shown consistently that a key factor behind atherogenesis within the arterial wall is the retention of low-density lipoprotein cholesterol (LDL-C) and other cholesterol-rich lipoproteins. Therefore, it is important for the prevention of cardiovascular diseases to lower the LDL cholesterol (4). European guidelines have recommended statins as first line therapy for reducing blood lipids since 1998 (5). Guidelines have focused on LDL-C targets for patients with different levels of CV risk, and these targets have been lowered considerably over time. Currently, LDL-C concentrations of < 2.6, 1.8, or 1.4 mmol/L are suggested as targets depending on the patients’ risk, as assessed by total coronary risk scores and other risk factors (6). A meta-analysis of randomized trials showed that statins can reduce the risk of major vascular events by about one-fifth and major coronary events by a quarter with each 1.0 mmol/L reduction of LDL-C (7). Statin utilization in Europe has increased over the years, but to varying degrees in different countries (8). Lithuania is one of the European countries with the lowest use of statins (8). Recently, Makarevicius et al. showed that the statin use has increased dramatically in Lithuania during the last decade (9), but there are no recent cross national comparisons of associations between trends in IHD mortality and statin utilization in the countries.

In the present study, we compared trends in statin utilization and IHD mortality in Lithuania and Sweden between 2000 and 2020. We specifically investigated how the utilization of different statin treatment intensities has changed in the two countries, and if there was any correlation between the total utilization of statins and IHD mortality.

## Methods

This was an ecological study comparing trends of statin utilization and IHD mortality rates between 2000 and 2020 in Lithuania and Sweden. These two countries have similar opportunities of providing high-quality data but have populations with differing cardiovascular risk profiles (1).

IHD mortality data were extracted from the Health Statistics Portal of the Institute of Hygiene, a Lithuanian budgetary institution under the Ministry of Health, and from The National Board of Health and Welfare in Sweden (10). Mortality data in both countries originate from death certificates, primary control of data entry is conducted, and completeness of data is controlled versus formats and defined logics (10, 11). The causes of death were selected using ICD-10 codes for ischemic heart diseases (I20–I25), including angina, acute myocardial infarction, subsequent myocardial infarction, and other ischemic heart diseases. All ages were included.

Aggregated data on statin sales in Lithuania during the study period were obtained from the IQVIA database that aggregates wholesale trade data and provides information on the manufacturer, trade names with dosages and package sizes for each month of the study period. Swedish statin utilization data were extracted from the Swedish Prescribed Drug Register, which contains information on all prescribed drugs filled at pharmacies based on ATC codes of different statins available throughout the study period (12). The utilization of statins was calculated in numbers of defined daily doses per 1000 inhabitants per day (DDD/TID), and also as numbers of packages dispensed for calculations of different statin intensities.

For Sweden, analyses were also made with individual level data to assess to what extent changes in volumes, measured as DDD/TID, corresponded to an increasing number of patients or to higher doses. For these analyses, period prevalence was used, calculated as the proportion of the population in the country each year dispensed at least one prescription with a statin.

For the analyses and comparisons in this study, the ATC/DDD Index 2023 was used for the study period and the ATC code for statins C10AA, as well as, ATC codes for individual statins – C10AA01 (simvastatin), C10AA02 (lovastatin), C10AA03 (pravastatin), C10AA04 (fluvastatin), C10AA05 (atorvastatin), and C10AA07 (rosuvastatin) as well as the combination of C10BA02 (simvastatin and ezetimibe), C10BA05 (atorvastatin and ezetimibe), and C10BA06 (rosuvastatin and ezetimibe) (13).

Moreover, statin sales were classified by their intensity of treatment to investigate how the use of low-, medium-, and high-intensity statins has changed over the years (Table 1) (13–15).

**Table 1:** Defined daily doses (DDDs) of statins and the definition of statin intensity (13–15).

Statin	DDD	Low intensity	Moderate intensity	High intensity
Atorvastatin	20 mg	-	10–20 mg	40–80 mg
Fluvastatin	60 mg	20–40 mg	80 mg ER	-
Lovastatin	45 mg	20 mg	40 mg	-
Pravastatin	30 mg	10–20 mg	40–80 mg	-
Rosuvastatin	10 mg	-	5–10 mg	20–40 mg
Simvastatin	30 mg	10 mg	20–40 mg	80 mg*

DDD: Defined daily doses.

* The highest dose of simvastatin is not recommended due to less favorable risk-benefit.

The age structures of the populations in Lithuania and Sweden are very similar. Therefore, statin utilization was not age-standardized, and IHD mortality was expressed as rates/100.000 inhabitants for comparisons between the countries.

Statistical analyses and calculations were performed using Statistical Package for the Social Sciences (SPSS) and Microsoft Excel 2016. To evaluate the strength of relationships between IHD mortality and statin utilization, correlation coefficients were calculated. In Sweden, Pearson’s correlation coefficient was used, but Lithuanian data were not normally distributed. Therefore, we used the Spearman’s rank correlation coefficient, which is calculated with the ranks of the values of each of the 2 variables instead of their actual values and thus differs from Pearson correlation (16). A *P* value of < 0.05 was considered statistically significant.

This study used aggregated data freely available in the public domain, with no sensitive information at the individual patient level. Therefore, ethical review board permission was not needed according to the legislation in the participating countries.

## Results

**Figure 1:**
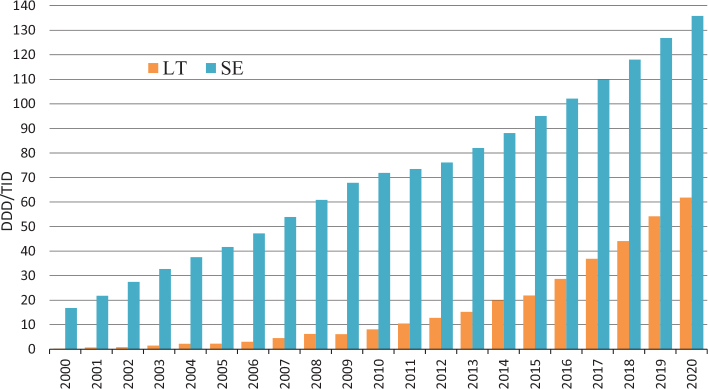
Utilization of statins in Lithuania (LT) and Sweden (SE) during 2000–2020. DDD/TID = Defined Daily Doses/1000 inhabitants.

The total utilization of statins increased over the study period in both countries but was considerably higher in Sweden throughout the study period (Figure 1). In 2000, the utilization of statins in Sweden was 16.8 DDD/TID, but in Lithuania, only 0.2 DDD/TID. In 2010, Sweden recorded a utilization of 71.9 DDD/TID that subsequently reached 135.8 DDD/TID in 2020. Meanwhile, Lithuania recorded an increase in statin use from only 8.1 DDD/TID in 2010 to 61.8 DDD/TID in 2020, i.e., still less than half of the utilization in Sweden.

**Figure 2:**
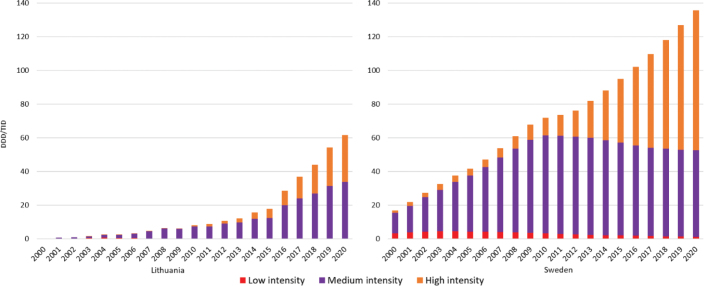
Utilization trends of different intensity statins in Lithuania and Sweden during 2000–2020. DDD/TID = Defined Daily Doses/1000 inhabitants.

When statins were classified by their dosage intensity (Table 1), it was revealed that medium intensity was the most common statin dosage in Lithuania during the whole study period (Figure 2). In 2000, low-intensity statins accounted for 40% of all statins sold, and the medium-intensity statins made up the remaining 60% of total statin utilization. After 2003, the proportion of low intensity compared to medium-intensity statins started decreasing. In 2008, medium-intensity statins made up almost 95% of all statins utilized (5.9 DDD/TID out of 6.2 DDD/TID). From 2009, the utilization of medium-intensity statins grew steadily, and they still made up the majority of all statins used (33.9 DDD/TID) in 2020. High-intensity statin utilization did not reach 1 DDD/TID during 2003–2010 but increased thereafter, reaching 27.9 DDD/TID (45% of all statins utilized) in 2020 (Figure 2).

Medium-intensity statins were the most commonly used statins, as assessed by DDDs, also in Sweden until 2016 (Figure 2). Overall, medium-intensity statins reached a peak utilization of 58.2 DDD/TID (out of 73.5 DDD/TID) and then decreased to 51.5 DDD/TID in 2020, making up around 38% of all statins dispensed that year. The utilization of high-intensity statins increased more markedly after 2011, and from 2017, the DDDs were greater for high- vs. moderate-intensity statins. In 2020, high-intensity statins comprised approximately 61% (83.1 DDD/TID) of all statins used (Figure 2).

**Figure 3:**
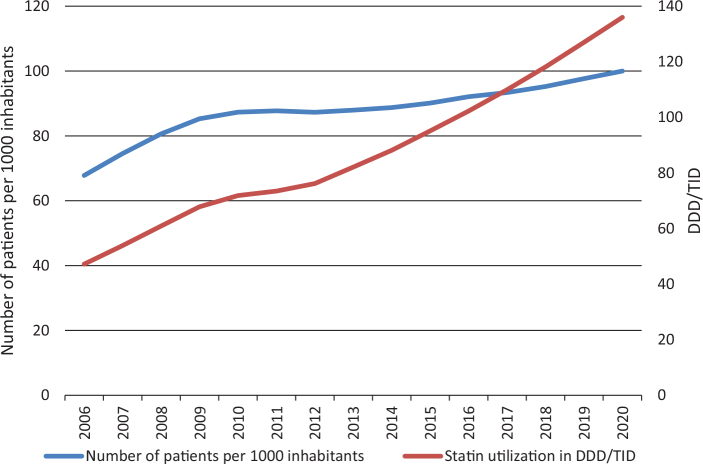
Number of patients per 1000 inhabitants treated with statins and total statin utilization expressed as DDD/TID in Sweden during 2006–2020.

Increasing statin utilization in DDD/TID based on sales data cannot simply be equated with more patients being treated as increased utilization of high dose treatment will account for part of the observed increases in volumes. This is shown in Figure 3 for the Swedish data; from approximately 2013, there is a steeper increase in the total statin DDDs/TID than in the numbers of patients treated due to the shift toward higher dosages.

**Figure 4:**
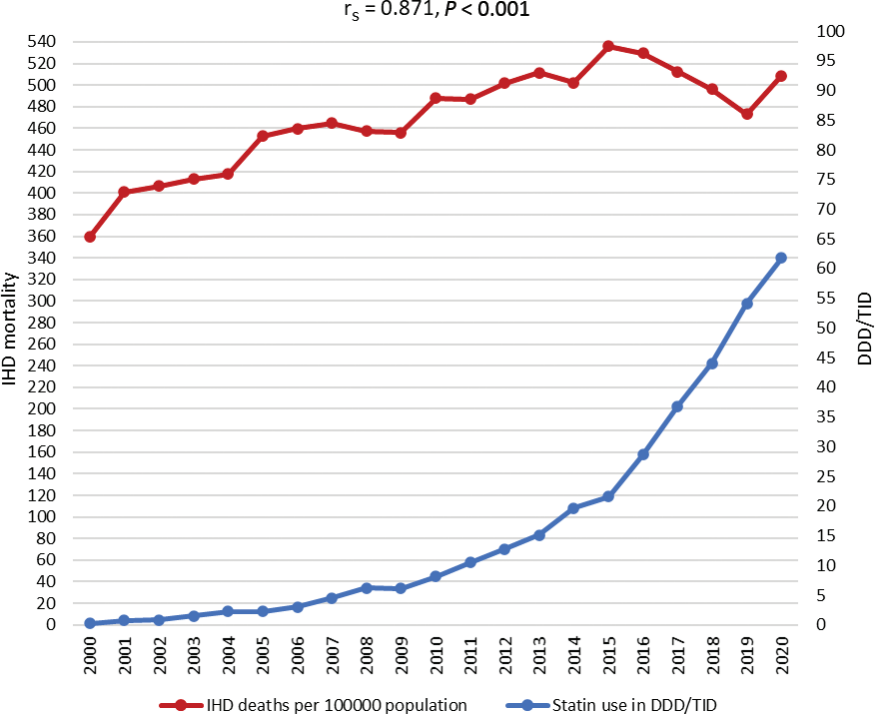
Ischemic heart disease mortality rates per 100.000 inhabitants and utilization of statins in Lithuania and Spearman’s rank correlation coefficient (rs).

IHD mortality in Lithuania remained very high throughout the study period (Figure 4). From the start of the millennium, there was a steady increase of IHD mortality until 2015 when the highest value of deaths was recorded – 535.8 deaths per 100.000 inhabitants. From 2000 to 2015, total statin utilization was below 22 DDD/TID. The period from 2015 to 2019 showed decreasing mortality from IHD, which coincided with a more rapid growth in statin utilization. However, despite this downward trend in mortality and growing statin utilization, Lithuania recorded a spike in deaths from IHD in 2020 again – 508.8 deaths per 100.000 inhabitants. When Spearman’s rank correlation coefficient was run to determine the relationship between IHD mortality and statin utilization in Lithuania, it showed value of 0.871 (*P* < 0.001), which demonstrated a strong positive correlation even though the ischemic heart disease mortality in Lithuania increased only slightly (Figure 4).

**Figure 5:**
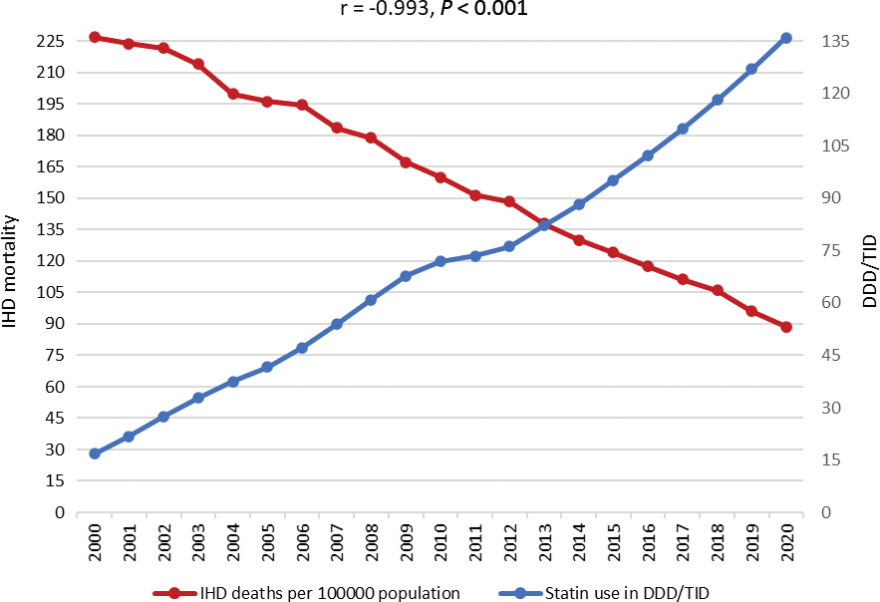
Heart disease mortality rates per 100 000 population and utilization of statins in Sweden and Pearson correlation coefficient (r).

In Sweden, a completely different situation was observed (Figure 5). IHD mortality decreased markedly during the study period (from 226.9 deaths per 100.000 inhabitants in 2000 to 88.7 deaths per 100.000 inhabitants in 2020). When the Swedish data on statin utilization and IHD mortality were tested using Pearson´s correlation coefficient, it indicated that IHD mortality was inversely related to the utilization of statins, and that this correlation is very strong (r = -0,993, *P* < 0.001).

## Discussion

In this study, by assessing trends in statin utilization and IHD disease mortality in Lithuania and Sweden, we found increased utilization of statins in both countries, but the associations between statin use and IHD mortality were totally different. While Sweden recorded steady and substantial decreases in IHD mortality over the years, Lithuania recorded an increased IHD mortality between 2000 and 2020. Moderate-intensity statin dosages were the most commonly used in both countries, but high-intensity statin treatment was introduced earlier and to a greater extent in Sweden.

The IHD mortality remained high in Lithuania throughout the years, in contrast to most other European countries showing substantial decreases in IHD (2). To some extent, this might be explained by ‘competing mortality risk’ with a decreasing mortality from accidents and injuries in Lithuania during the period, but there has been no major change in deaths due to cancer or other major causes of death, and this, therefore, most likely has no major impact on the findings (17).

Several factors may explain the different development of IHD mortality between the two countries. A review of studies on factors explaining the development of CHD mortality noted that improvement in life style factors was the key drivers to initiate decline, while further improvement was attributed to increasing proportions of patients receiving evidence-based treatments (18). Smoking, elevated cholesterol levels, diabetes, diet, sedentary lifestyle, hypertension, and obesity are well established modifiable risk factors for CVD (6). A longitudinal follow-up of a population in northern Sweden showed significant improvements in major cardiovascular risk factors over time (19). In elderly men and women, blood-pressure decreased 12.6/6.1 mmHg between 1994 and 2009, and the prevalence of smoking was halved between 1986 and 2009 (19). However, this study also showed that BMI increased, and the prevalence of diabetes remained stable over time. A study assessing cardiovascular risk factors among Lithuanian middle-aged adults participating in a primary prevention program between 2009 and 2018 revealed a high prevalence of all CVD risk factors in 2009 with slight decreases during the period in most prevalence rates, except for dyslipidemia and smoking (20). Dyslipidemia was the most common CVD risk factor in both genders and showed a very high prevalence (approximately 90%) between 2009 and 2018. During 2009–2018, systolic and diastolic blood pressures decreased among Lithuanian men and women, but the proportion of people with controlled hypertension only increased from 20.4% to 25.1%. Obesity rates declined among women, while they remained constant among men, and the prevalence of metabolic syndrome also declined among women but increased among men (20). Better management of these risks factors appears to be very important to lower the IHD mortality in Lithuania. A survey of trends in Norway showed that changes in coronary risk factors accounted for a total of 66% of the decline in total coronary heart disease between 1994 and 2008 (21). The largest single contributor was declining cholesterol, which contributed 32% to the decline, whereas blood pressure, smoking, and physical activity each contributed 14%, 13%, and 9%, respectively. Increases in body mass index and the prevalence of diabetes mellitus were associated with 7% and 2% increases in the risk of CHD, respectively, in the Norwegian study.

There may also be other underlying important differences in the cardiovascular risk of the population in Sweden and Lithuania. Twenty years ago, the LiVicordia study compared risk factors for CVD among 50-year-old men in Linköping, Sweden, and Vilnius, Lithuania (22). While small differences were found in traditional risk factors between the two cohorts, important differences related to oxidative and psychosocial stress were identified, which may contribute to different trajectories of development of CVD and subsequent mortality.

The different uptakes of statins, in particular high dose statins, in the two countries, may have contributed to the differences in IHD mortality trends over time. Statin utilization in Lithuania increased substantially only during recent years, and we know from randomized clinical trials that the benefit of statin treatment is small during the first year of treatment but accumulates thereafter (23). Consequently, the lack of declining IHD mortality in Lithuania could at least partially be explained by the fact that patients were not receiving the appropriate treatment to reach lipid-lowering therapy goals. The statin utilization was very low during many years despite a very high prevalence of dyslipidemia, and the vast majority of statins used in Lithuania throughout the study period was of moderate intensity. High-intensity statin utilization became more prominent only in recent years when a minor decline in mortality was observed. Furthermore, the Dyslipidemia International Study completed in Lithuania, Latvia, and Estonia revealed that many patients treated with statins did not meet the LDL-C and other lipid targets (24). Thus, 80.7% of all patients in the study had elevated LDL-C despite being on statin treatment, and in the very high CVD risk group, 86.5% of the patients did not attain their LDL-C goals. Similar trends were observed with other lipids, as 26% of patients had low HDL-C and 35% had elevated triglycerides.

In Sweden, moderate-intensity statins also comprised the majority of all statins dispensed until 2016. From 2017, high-intensity statins became the most utilized ones in terms of DDDs. However, a cross-sectional register study performed in a primary care setting in Sweden found that of the 37120 patients in the secondary prevention, only 18% reached the recommended LDL-C goal of ≤1.8 mmol/L, and one-third of patients with CHD were not even on lipid-lowering treatment (25). Based on individual risks, the estimated number of CVD events in the study population might be reduced by 14% if all patients without a statin or with less potent statin treatment were given atorvastatin 80 mg. The pattern of statin utilization may be important as overuse in patients with low cardiovascular risk and underuse in patients with high cardiovascular risk have been noted (26, 27). This is important to evaluate as the absolute risk reduction is greater in secondary prevention than in primary prevention (23). This study did not assess whether increases in total statin utilization in Sweden and Lithuania were attributable to secondary or primary prevention. This might contribute to the different trends in IHD mortality recorded in Sweden and Lithuania despite increasing statin utilization – statins seem to be more underutilized in high-risk patients in Lithuania.

The slow growth of statin utilization until 2015 in Lithuania is most likely related to very strict reimbursement rules. From 2008 to 2015, statins were reimbursed only for secondary prevention and for patients in a high CVD risk group with total TC ≥7.5 mmol/L, LDL-C ≥ 6.0 mmol/L, or TG ≥ 4.5 mmol/L. Additionally, from 2000 to 2015, statins could only be prescribed by cardiologists. At the end of 2015, new reimbursement rules were introduced, and the right to prescribe statins was no longer limited to cardiologists. Reimbursement of 80% became available for patients with LDL-C ≥ 3.0 mmol/L and for patients with very high-risk if their LDL-C was ≥1.8 mmol/L. In 2019, 100% reimbursement for statins became available for patients at very high-risk (LDL-C ≥ 1.8 mmol/L), high-risk (LDL-C ≥ 3.0 mmol/L), and intermediate-risk (LDL-C ≥ 3.0 mmol/L), as well as for people with a family history of premature coronary artery disease (LDL-C ≥ 5.0 mmol/L). In Sweden, simvastatin was fully covered by the reimbursement scheme during the whole period. From 2009, reimbursement atorvastatin and rosuvastatin were restricted to patients not reaching treatment targets with generic statins (28). Atorvastatin was fully reimbursed again after patent expiry in 2013 and rosuvastatin in 2018.

Many studies have shown that persistence and adherence to statin therapy may be very low, and that patients tend to discontinue their treatment early (29). For example, 63% of patients discontinued their statin treatment between 2009 and 2016, and the median time to first discontinuation was 1.5 years in a Scottish cohort study (30). Such early discontinuation is especially worrisome since the proportional risk reduction is much smaller in the first year of statin treatment, and long-term use of statins is important to achieve the benefits of lipid-lowering treatment (23). A recent observational study of all adult patients who had suffered a myocardial infarction or received coronary revascularization during 2012 to 2018 in the region of Stockholm, Sweden, and initiated lipid-lowering therapy showed that 91% of the patients were adherent (PDC ≥ 80%) at baseline and 70% remained adherent to therapy even after 7 years (31). Out of 20.490 patients, only 2% discontinued their treatment permanently. Patients with poor adherence (PDC < 80%) and those who discontinued the treatment were at higher risk of major adverse cardiovascular events. Interestingly, good adherence appeared to be more important than use of high-intensity statin treatment for beneficial outcomes (31). There is no such study from Lithuania, but a study on the persistence to antihypertensives showed that 57% of all patients initiated on treatment had discontinued the treatment after 1 year (32). This indicates that persistence and adherence may be much poorer in Lithuania, and this could be one of the reasons why mortality did not decrease in Lithuania like it did in Sweden when statin utilization increased.

Lithuania recorded a spike in deaths from IHD in 2020 despite growing statin utilization and a downward trend in mortality from 2015 to 2019. However, it is important to recognize that 2020 was an extraordinary year after the outbreak of the coronavirus disease pandemic (33). Several cardiometabolic risk factors were associated with hospitalization or death due to COVID-19 (34). There is also evidence that statins reduce mortality in patients with COVID-19 infection (35). However, the intensified focus on COVID-19 prevention and treatment, as well as lockdown measures and physical distancing restrictions, impacted healthcare services and patient care negatively (36). It is possible that this may have been the case in Lithuania. However, a similar effect was not observed in Sweden, where the IHD mortality continued to decrease during the pandemic (37). Obviously, the interplay among the pandemic, statin utilization, and IHD mortality is complex and requires further research.

### Strengths and limitations

The findings of this study have to be interpreted within its strengths and limitations. A major strength is the duration of the study with analyses of the total statin utilization and IHD mortality during 20 years. The analyses were based on complete and good quality covering 100% of the populations in Sweden and Lithuania regardless of their socioeconomic or reimbursement status. Additionally, this study compared statin utilization and IHD mortality in two countries with different epidemiological situations, which is rarely done. Moreover, we studied the utilization of different statin dosages, and how they changed over time. The use of DDD/TID as a measure of statin utilization makes further international comparisons feasible.

This study also has several limitations. First, all drug utilization data were obtained in aggregated form, and no individual patient level information (age, sex or gender, indications, duration of the treatment, etc.) was obtained and used to describe the time trends. Also, the use of aggregate data in Lithuania does not allow us to determine whether the increased statin use was a result of a higher incidence or better persistence. As the Swedish database revealed that the number of patients treated did not increase as much as the total statin utilization, it would be very valuable to have such data available in Lithuania, too. Second, the use of DDDs could also be considered a limitation. The DDD values assigned to statins are low, especially since the use of more potent, higher intensity statin treatment is now preferred, and this can lead to a substantial overestimation of statin utilization. It is also important to acknowledge that pharmacy fills or wholesalers’ sales of statins do not mean that patients actually took the medicine. Moreover, the analysis of statin utilization in Lithuania was based on data from wholesalers’ sales, which could slightly overestimate the amount of statins used in Lithuania. Finally, the mortality data were not age standardized in the country comparisons. We anticipate that this would have a minor impact on the overall findings, but the differences in cardiovascular risk factors and psychological stress between the two populations may have implications for the overall life expectancy (22, 38).

In summary, statin utilization and IHD mortality differed markedly over time between Sweden and Lithuania. The larger and more rapidly increasing statin utilization in Sweden was closely associated with a continual reduction of IHD mortality, whereas the IHD mortality in Lithuania tended to increase from a much higher level despite increasing statin utilization. Restrictions on statin prescribing, reimbursement in Lithuania up to 2015, a delayed adoption of high-intensity treatment, and a higher burden of CV risk factors in Lithuania, as well as possible differences in persistence and adherence to statin treatment may all have contributed to the differences observed in this study. Improved CV risk factor management and increased statin utilization should be priorities in Lithuania.

## Disclosure statement

The authors declare no conflict of interest.

## Notes on contributors

BW and PH were responsible for the study conception and design. Data management, data collection, and data analysis were performed by IT and KD. The first draft of the manuscript was written by KD based on a prior master thesis written by KD, under the supervision of BW. All authors commented on different versions of the manuscript as well as read and approved the final manuscript.

## ORCID

Indre Treciokiene https://orcid.org/0000-0001-6583-9999

Paul Hjemdahl https://orcid.org/0000-0001-5346-275X

Björn Wettermark https://orcid.org/0000-0003-0531-2516
